# Prevalence of cardio-metabolic risk factors in a nationally representative sample of Iranian adolescents: The CASPIAN-III Study

**DOI:** 10.15171/jcvtr.2017.02

**Published:** 2017-03-13

**Authors:** Roya Kelishadi, Ramin Heshmat, Farshad Farzadfar, Mohammad Esmaeil Motlag, Maryam Bahreynian, Saeid Safiri, Gelayol Ardalan, Ehsan Rezaei Darzi, Hamid Asayesh, Fatemeh Rezaei, Mostafa Qorbani

**Affiliations:** ^1^Department of Pediatrics, Child Growth and Development Research Center, Research Institute for Primordial Prevention of Noncommunicable Disease, Isfahan University of Medical Sciences, Isfahan, Iran; ^2^Chronic Diseases Research Center, Endocrinology and Metabolism Population Sciences Institute, Tehran University of Medical Sciences, Tehran, Iran; ^3^Non-Communicable Diseases Research Center, Endocrinology and Metabolism Population Sciences Institute, Tehran University of Medical Sciences, Tehran, Iran; ^4^Department of Pediatrics, Ahvaz Jundishapur University of Medical Sciences, Ahvaz, Iran; ^5^Managerial Epidemiology Research Center, Department of Public Health, School of Nursing and Midwifery, Maragheh University of Medical Sciences, Maragheh, Iran; ^6^Department of Medical Emergencies, Qom University of Medical Sciences, Qom, Iran; ^7^Department of Social Medicine, School of Medicine, Jahrom University of Medical Sciences, Jahrom, Iran; ^8^Non-Communicable Diseases Research Center, Alborz University of Medical Sciences, Karaj, Iran

**Keywords:** Cardio-metabolic Risk Factors, Liver Enzymes, Socioeconomic Status, Adolescents

## Abstract

***Introduction:*** The aim of the present study is to explore the prevalence and mean of cardiometabolic risk factors and liver enzymes of Iranian adolescents living in regions with different socioeconomic status (SES). To the best of our knowledge this is the first study reporting these data at sub-national level in Iran.

***Methods:*** This multi-centric study was performed in 2009-2010 on a stratified multi-stage probability sample of 5940 students aged 10-18 years, living in urban and rural areas of 27 provinces of Iran. Trained healthcare professionals measured anthropometric indices, systolic and diastolic blood pressures (SBP, DBP) according to standard protocols. Fasting venous blood was examined for fasting blood sugar (FBS), lipid profile and liver enzymes including alanine aminotransferase (ALT) and aspartate aminotransferase (AST). We classified the country into four sub-national regions based on criteria of the combination of geography and SES. Mean and frequency of risk factors were compared across these regions.

***Results:*** The mean of body mass index had linear rise with increase in the regions’ SES (*P * for trend <0.001). The mean levels of DBP, total cholesterol (TC), high-density lipoproteincholesterol (HDL-C), triglycerides (TG), FBS, ALT, and AST had linear association with regions’ SES in the whole population and in both genders (*P * for trend < 0.05), whereas the corresponding figure was statistically significant for the mean SBP only in girls (*P * for trend: 0.03) and for the mean of LDL-C in the whole population and in boys (*P * for trend <0.001). In total and in both genders, there was an escalating trend in the prevalence of elevated FBS, TC and liver enzymes, low HDL-C, and metabolic syndrome by increase in the SES of the region(*P * for trend <0.01).

***Conclusion:*** This study proposes that in addition to national health policies on preventing cardiometabolic risk factors, specific interventions should be considered according to the regional SES level.

## Introduction

High prevalence of non-communicable diseases (NCD) and related disorders particularly cardiovascular disease (CVD), metabolic syndrome (MetS), obesity, type II diabetes and lipid disorders are a main health concern in all over the world.^1,2^ It is well documented that the etiology of cardio-metabolic risk factors is multifactorial and is based on the interaction between genetics and environment, lifestyle behaviors, and socioeconomic status (SES).^3,4^

High frequency of cardio-metabolic disorders have been documented in some previous studies, for instance 20.2% of American youths had an unfavorable concentration of total cholesterol (TC), high-density lipoprotein-cholesterol (HDL-C), or non-HDL-C and 11.0% had either high or borderline blood pressure (BP).^5^ In addition, an approximately 20% of 8-17 years children and adolescents had an adverse lipid profile including TC, HDL-C, or non-HDL-C and more than 10% had borderline high or high BP.^5^ A study on Chinese children , aged 7 to 17 years, showed that the prevalence of hypertension, dyslipidemia, and hypertriglyceridemia were higher in those with severe obesity than in those with moderate obesity.^6^

The prevalence rate of obesity is increasing worldwide; the frequency of overweight and obesity increased from 31.7% to 33.4%, during the year 2002 to 2006. The overweight prevalence increased from 16.8%-17.9% among 13-year-old Italian adolescents, from 2004 to 2006; and from 13.3% to 19.7% among 15-year-old teenagers.^7^ In Iran, we previously indicated the prevalence rate of general and abdominal adiposity as 11.89% (13.58% of boys vs. 10.15% of girls) and 19.12% (20.41% of boys vs. 17.79% of girls), respectively.^8^ We also showed that excess weight was more prevalent in high SES regions, whereas underweight and short stature were more observed in low SES districts of the country.^9^

The epidemic of NCDs is now occurring in low- and middle-income countries; this is of special interest for the Middle East and North Africa (MENA) population with the highest comparative prevalence of type II diabetes in the world.^10^ Epidemiologic transition and rapid economic growth are considered as the main reasons of this emerging problem. As one of the countries of this region, Iran has high frequency of NCDs, cardio-metabolic risk factors, and non-alcoholic fatty liver disease.^11-13^

As chronic diseases risk factors track from childhood to adult life,^14^ prevention, screening, and early management of them might help tailoring intervention strategies to prevent excess burden of NCDs. Nationwide studies in Iran revealed high prevalence of cardio-metabolic risk factors among the Iranian pediatric population.^15,16^ For instance, in our previous study, according to the criteria of the International Diabetes Federation (IDF) for the adolescent age group, the prevalence of having one, two, three and all four components of MetS was 24.2% , 8.0%, 2.1%, and 0.3%, respectively.^11^ However no previous study has reported this situation at sub-national regions of Iran, as a vast country with large variations in health and SES in different regions.

The present study aims to determine the prevalence and mean of cardio-metabolic risk factors and liver enzymes in a nationally- representative sample of Iranian adolescents living in regions with different SES.

## Materials and Methods

Data of the present study were collected as a part of the “national survey of school student high risk behaviors” (2009-2010) named as the third survey of the school-based surveillance system entitled Childhood and Adolescence Surveillance and PreventIon of Adult Non-communicable disease (CASPIAN-III) Study. This school-based nationwide health survey was conducted in 27 provinces in Iran in 2009-2010. Details on the study protocol have been described before,^17^ and here we report it in brief.

Study protocols were reviewed and approved by ethics committees and other relevant national regulatory organizations. Before recruitment to the study, a complete description of the study protocol and its purpose was sent to parents. Signatures of parents on written informed consent form and verbal assent from students were obtained.

### Measurements

The questionnaires were prepared in Farsi based on and the World Health Organization (WHO) Global School Health Survey, and added some more questions to the parental questionnaire. The questionnaires had items about family dietary habits, students’ past history, and family history of chronic non-communicable diseases. The content validity of the questionnaires were confirmed based on the experts’ panel opinions and item analysis. Reliability measures were evaluated in a pilot study.^17^

The physical examination had been conducted by a team of expert physicians, nurses, and healthcare professionals according to the standard protocols, and by applying calibrated instruments. Waist circumference (WC), weight and height were measured, and body mass index (BMI) was calculated as weight (kg) divided by height squared (m^2^).

Systolic and diastolic blood pressure (SBP and DBP) were measured under standard protocol by using calibrated mercury sphygmomanometers and appropriate size cuff.^18^ Fasting venous blood sample was examined for fasting blood glucose, lipid profile, and liver enzymes.^17,19^

### Definition of risk factors

Overweight and obesity were defined according to the growth curves of the WHO i.e., overweight as gender- specific BMI for age of > +1 *z*-score, and obesity as gender-specific BMI of > +2 *z*-score.^20^

High fasting blood sugar (FBS) was considered according to the recommendation of the American Diabetes Association, i.e. equal or greater than 100 mg/dL.^21^

Abnormal lipid levels were defined according to the guideline of the National Heart, Lung, and Blood Institute (NHLBI) for cardiovascular health and risk reduction in children and adolescents. Based on these cut points, the following levels were considered as risk factor: TC ≥200 mg/dL, low-density lipoprotein cholesterol (LDL- C) ≥130 mg/dL , triglycerides (TG) ≥130 mg/dL, and HDL-C <40 mg/dL.^22^

High BP was considered as SBP and/ or DBP levels equal or more than the age- and gender- specific ≥95th percentile.^18,22^

MetS was defined according to the criteria of the International Diabetes Federation for the pediatric age group, i.e. the coexistence of at least three of the following components: FBS ≥100 mg/dL, TG ≥150 mg/dL, HDL-C <40 mg/dL, waist circumference > 90th percentile, or elevated BP (SBP >130 mm Hg, or DBP >85 mm Hg).^23^

Elevated liver enzymes were considered as alanine aminotransaminase (ALT) or aspartate aminotransaminase (AST) greater than 40IU.^19^

The country was categorized into four sub-national regions.^24^ The sub-national regions were identified according to the criteria of the combination of geography and SES using principal component analysis (PCA). According to this classification, the southeast and the central region have the lowest and highest SES, respectively. SES was an index including variables from the 2006 census, such as literacy, family permanent income and employment rate.^23^

### Sampling procedure, sample size and statistical analysis

The sample size was calculated based on different variables but the maximum sample size was considered. This figure was calculated to be 4950 in the 27 provinces of Iran, and with estimated 80% response rate, 20% was added to the sample size (n = 5940). The study participants were 5682 Iranian school students aged 10 to 18 years. They were selected from elementary, intermediate, and high school of rural and urban areas of 27 provinces of Iran through stratified multistage random sampling method. Stratification was done according to the school type (public/private) and location (urban/rural). From each stratum, proportional 2-stage cluster sample of students were selected. Iranian nationality was the inclusion criterion for the study, and all foreign-nation students were not included. Moreover, those students with history of chronic or acute disease and any medication use were not included in this study. A total of 5682 students who filled out completely the questionnaire were included in the analysis of this article. Categorical variables are expressed as number, percentages and continuous variables are presented as mean, standard deviation (SD). Comparison of mean and prevalence of various cardio-metabolic risk factors and liver enzymes across regions were investigated by analysis of variance (ANOVA) and chi-square test respectively. Design of sampling method was considered in analysis using survey data analysis method. All graphs were depicted using R package (version 3.2.1, R Foundation for Statistical Computing, Vienna, Austria) and all statistical analyses were performed using Clarify programs installed in the Stata statistical software package (Release 9. College station, TX: StataCorp LP.). Missing data were considered with Amelia package in R software (version 3.2.1, R Foundation for Statistical Computing, Vienna, Austria). Clarify is a program that uses stochastic Monte-Carlo simulation for estimating mean and prevalence. A *P* value of less than 0.05 was considered as statistically significant.

## Results

**Figure 1:**
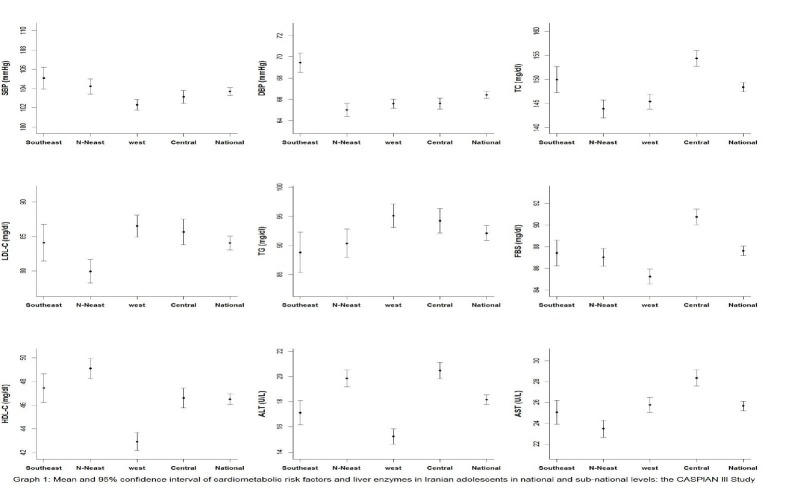
Mean and 95% confidence interval of cardiometabolic risk factors and liver enzymes in Iranian adolescents in national and subnational levels: the CASPIAN III study.

**Figure 2:**
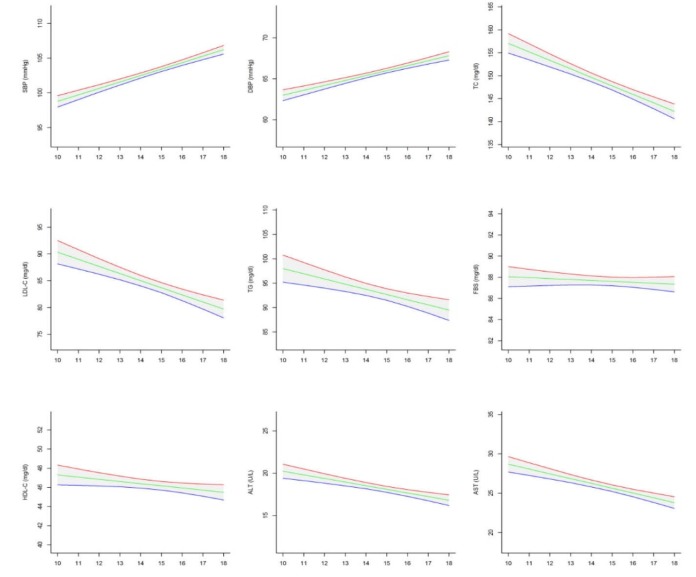
Association between age and cardiometabolic risk factors and liver enzymes in Iranian adolescents: the CASPIAN III study.

**Figure 3:**
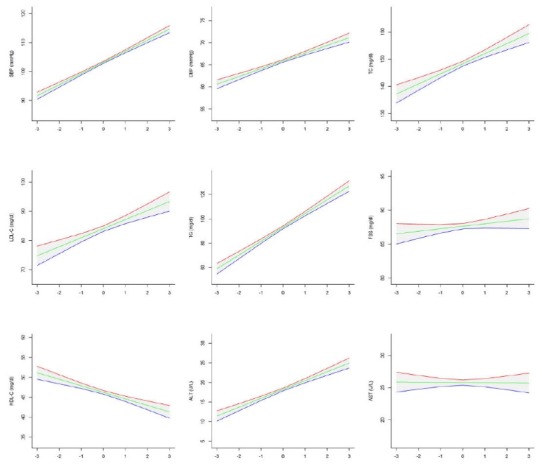
Association between BMI z-score and cardiometabolic risk factors and liver enzymes in Iranian adolescents: the CASPIAN III study

Out of the 5940 invited students, 5682 completed the study (response rate of 95.6%). The frequency of students in Southeast (lowest SES), North-Northeast (second low SES rank), West (second high SES rank) and Central (highest SES) regions were respectively as follows; 567 (10.0%), 1075 (18.9%), 2372 (41.7%) and 1668 (29.4%). No significant difference was found in age and sex ratio across the regions (*P* value >0.05). Table 1 shows the anthropometric indexes, body mass index and waist circumference (BMI and WC), age and sex of participants in national and sub-national levels. The mean of BMI was linearly increased with increasing the regions’ SES (*P* for Trend <0.001), whereas the corresponding figure was not significant for the mean of WC (*P* for Trend >0.05). The association of age, WC and BMI Z-score with cardio-metabolic risk factors and liver enzymes in national level are presented in Figure 1-3 respectively.

**Table 1:** Baseline characteristics of Iranian adolescents in national and sub-national levels: the CASPIAN III Study

**Variable**	**Regions**					
	**Southeast**	**North- northeast**	**West**	**Central**	**National**	***P*** **for trend** ^2^
	**Mean (SD)** ^1^	**Mean ( SD )** ^1^	**Mean ( SD )** ^1^	**Mean ( SD )** ^1^	**Mean ( SD )** ^1^	
WC (cm)	68.68 (33.08)	68.24 (20.64)	68.26 (21.51)	69.71(12.35)	68.72(20.67)	0.132
BMI (kg/m^2^)	18.46 (3.81)	19.59 (4.23)	19.26 (3.90)	19.86(4.30)	19.42(4.10)	<0.001
Age (years)	14.49 (2.58)	14.71 (2.36)	14.78(2.38)	14.72(2.40)	14.72 (2.40)	0.112
Sex (boy)	288 (50.8)	539 (50.1)	1209 (51.0)	811 (48.6)	2847 (50.1)	0.351

Abbreviations: SE: standard error of mean, CI: confidence interval, WC: waist circumference, BMI: body mass index. ^1^For sex data are presented as N (%). ^2^ *P* values are resulted from ANOVA test except for sex which is resulted from chi-square test.

**Figure 4:**
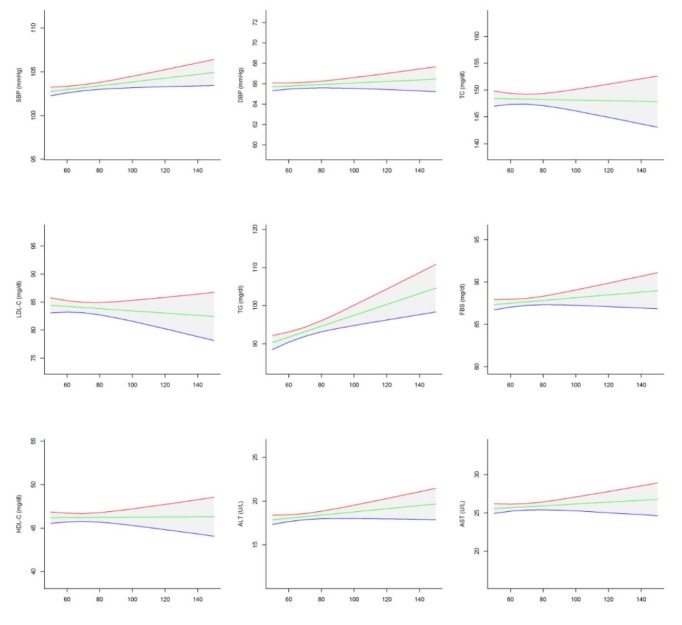
Association between WC and cardiometabolic risk factors and liver enzymes in Iranian adolescents: the CASPIAN III study.

Mean and 95% CI values of various cardio-metabolic risk factors and liver enzymes in national and sub-national levels are presented in Table 2 and Figure 4. The means of DBP, TC, HDL, TG, FBS, ALT, AST and LDL/HDL ratio was linearly associated with regions’ SES in boys, girls and overall (*P* for Trend <0.05), whereas the corresponding figure for the mean of SBP only in girls (*P* for Trend: 0.03) and for the mean of LDL only in boys and overall was statistically significant (*P* for Trend <0.001). The mean of SBP, HDL-C and ALT was significantly lower in the West region (second high SES rank) than in other regions (*P* value< 0.001), whereas the mean of LDL-C was significantly lower in the North-Northeastregion (second low SES rank) than in other regions (*P* value< 0.001). The mean of TC, FBS and AST in Central region (highest SES), TG in the West region (second high SES rank), and DBP in the Southeast region (lowest SES) was significantly higher than in other regions. The average of SBP, DBP, FBS, ALT and AST was significantly higher in girls, whereas the mean of TC and LDL-C was significantly higher in boys (*P*-value< 0.001). No significant difference was found in HDL and TG between both sexes (*P* value >0.05).

**Table 2:** Mean (SD) of cardiometabolic risk factors and liver enzymes in national and sub-national levels: the CASPIAN III Study

**Variable**	**Regions**					***P*** ** for trend** ^1^
	**Southeast**	**North-Northeast**	**West**	**Central**	**National**	
	**Mean (SD)**	**Mean (SD)**	**Mean (SD)**	**Mean (SD)**	**Mean (SD)**	
SBP (mm Hg)											
Boys	102.0 (14.35)	103.2 (12.89)	100.4 (14.12)	101.6 (12.91)	101.43(13.65)	0.067
Girls	108.7 (13.09	105.2 (13.67)	104.4 (13.71)	104.5 (14.62)	104.98(13.96)	0.03
DBP (mm Hg)											
Boys	67.89 (10.58)	64.82 (10.54)	63.69(10.70)	65.22 (9.82)	64.76(10.49)	0.050
Girls	71.45 (10.63)	65.19 (11.38)	67.71 (10.89)	95.95 (11.02)	66.97(11.11)	0.002
TC (mg/dL)											
Boys	152.9 (30.98)	145.3 (29.85)	147.6 (29.27)	158.2 (34.15)	150.84(31.54)	<0.001
Girls	146.5 (26.93)	142.5 (30.94)	143.0 (29.92)	150.7 (34.84)	145.74(31.69)	<0.001
HDL											
Boys	48.18 (12.79)	49.34 (15.62)	43.04 (12.67)	47.13 (14.52)	46.55(14.24)	<0.001
Girls	46.61 (12.86)	48.84 (17.45)	42.77 (11.65)	46.09 (13.68)	45.86(14.30)	0.001
LDL -C (mg/dL)											
Boys	85.37 (25.37)	80.45 (27.64)	88.27 (25.42)	88.94 (28.77)	85.88(27.18)	<0.001
Girls	82.61 (22.07)	79.44 (28.00)	84.58 (26.73)	82.26 (29.30)	82.23 (27.34)	0.123
TG (mg/dL)											
Boys	90.64 (42.29)	91.55 (32.93)	96.52 (42.58)	93.67 (43.38)	93.80(40.83)	0.041
Girls	86.67 (41.71)	89.11 (36.48)	93.58 (43.35)	94.81 (50.39)	92.18(44.29)	<0.001
FBS (mg/dL)											
Boys	87.43 (17.69)	86.97 (17.56)	84.81 (12.21)	89.16 (13.14)	86.97(14.71)	0.051
Girls	87.41 (11.56)	87.05 (11.27)	85.67 (12.58)	92.36 (13.49)	88.30(12.77)	<0.001
ALT (U/L)											
Boys	16.81 (12.13)	18.64 (10.79)	14.48 (9.82)	20.01 (12.41)	17.41(11.41)	0.008
Girls	17.49 (10.33)	21.03 (10.89)	16.03 (10.96)	20.88 (12.65)	18.97(11.63)	0.023
AST (U/L)											
Boys	23.42 (7.97)	22.01 (11.14)	24.87 (13.05)	26.87 (18.30)	24.48(13.85)	0.008
Girls	26.88 (9.22)	24.93 (10.59)	26.71 (11.15)	29.73 (18.53)	27.15(13.49)	<0.001
LDL/HDL											
Boys	1.87 (0.71)	1.83 (0.86)	2.29‏ (1.03)	1.96 (0.88)	2.01 (0.93)	<0.001
Girls	1.88 (0.72)	1.82 (0.82)	2.15 (0.94)	1.99 (1.04)	1.97 (0.92)	<0.001
ALT/AST											
Boys	0.75 (0.45)	0.92 (0.38)	0.62 (0.36)	0.84 (0.47)	0.78 (0.43)	0.498
Girls	0.69 (0.41)	0.91 (0.41)	0.62 (0.40)	0.78(0.45)	0.75 (0.44)	0.193

Abbreviations: SD: standard deviation of mean, CI: confidence interval, SBP: systolic blood pressure, DBP: diastolic blood pressure, TC: total cholesterol, HDL: high-density lipoprotein, LDL: low-density lipoprotein, TG: triglyceride, FBS: fasting blood sugar, ALT: alanine aminotransferase, AST: aspartate aminotransferase, BMI: body mass index, WC: waist circumference. ^1^P-values are resulted from ANOVA test.

Table 3 demonstrates the prevalence of the various cardio-metabolic risk factors and elevated liver enzymes in national and sub-national levels. It was observed that in overall and in both sexes, the prevalence of elevated FBS, elevated TC, low HDL-C, elevated AST and MetS was linearly increased with increasing the regions’ SES (*P* for Trend <0.01). The corresponding figure was not significant for SBP (*P* for Trend >0.05). Overall and in girls, a linear association was observed between the prevalence of elevated TG, elevated LDL-C, elevated DBP with regions’ SES, whereas this linear association was not observed for elevated ALT in girls (*P* for Trend >0.05).

**Table 3:** Prevalence of cardiometabolic risk factor and liver enzymes in adolescents in national and sub-national levels: the CASPIAN III Study

Variable	**Regions**					***P*** **for trend** ^1^
	**Southeast**	**North-Northeast**	**West**	**Central**	**National**	
	**No. (%)**	**No. (%)**	**No. (%)**	**No. (%)**	**No. (%)**	
High FBS						
Boys	22 (7.8)	22 (7.8)	78 (10.2)	135 (19)	280(12.18)	<0.001
Girls	36 (14.1)	36 (14.1)	108 (14.4)	198 (28.9)	410(18.45)	<0.001
High TG ≥150 mg/dL						
Boys	25 (8.9)	25 (4.7)	78 (9.6)	62 (8.7)	190 (8.1)	0.164
Girls	13 (5.1)	29 (5.5)	62 (8.1)	77 (10.4)	181(7.9)	<0.001
High TG ≥130 mg/dL						
Boys	42 (14.6)	66 (12.2)	133 (16.3)	106 (14.9)	347(14.8)	0.444
	26 (10.2)	46 (8.7)	124 (16.3)	119 (16.1)	315(13.8)	<0.001
High TC						
Boys	20 (7.1)	17 (3.2)	39 (4.5)	77 (10.8)	153(6.4)	<0.001
Girls	6 (2.4)	20 (3.8)	31 (3.8)	61 (8.2)	118(5.1)	<0.001
High LDL						
Boys	14 (6.8)	21 (4.4)	36 (6.3)	34 (7.9)	105(6.2)	0.160
Girls	5 (2.6)	21 (4.5)	33 (6.1)	27 (6.6)	86(5.3)	0.024
Low HDL						
Boys	66 (23.5)	139 (25.8)	280 (42.9)	191 (35.3)	676(33.6)	<0.001
Girls	73 (28.6)	179 (33.6)	273 (42.9)	212 (37.2)	737(36.9)	0.004
High ALT						
Boys	13 (4.6)	9 (1.7)	8 (1.22)	39 (6.9)	69(3.39)	0.007
Girls	10 (3.9)	18 (3.4)	15 (2.4)	34 (5.9)	77(3.81)	0.118
High AST						
Boys	10 (3.5)	15 (2.8)	51 (7.1)	64 (11.3)	140(6.7)	<0.001
Girls	16 (6.3)	26 (4.9)	51 (7.4)	70 (11.9)	163(7.9)	<0.001
High SBP						
Boys	9 (3.4)	21 (4.1)	27 (2.3)	16 (2.2)	73(2.7)	0.066
Girls	13 (5.6)	22 (4.3)	49 (4.7)	41 (5.2)	125(4.9)	0.854
High DBP						
Boys	8 (2.9)	10 (1.9)	25 (2.1)	18 (2.4)	61(2.2)	0.890
Girls	22 (9.6)	18 (3.4)	47 (4.4)	27 (3.3)	114(4.3)	0.004
High SBP and/or DBP						
Boys	17 (6.4)	27 (5.4)	46 (4.0)	31 (4.3)	121(4.6)	0.122
Girls	27 (12.5)	33 (6.6)	82 (8.1)	54 (7.0)	196(7.8)	0.099
MetS						
Boys	5 (1.9)	10 (2.0)	19 (3.2)	20 (4.5)	54 (3)	0.020
Girls	6 (3.1)	12 (2.4)	25 (4.4)	45 (9.9)	88 (5.2)	<0.001

Abbreviations: High fasting blood sugar (FBS) ≥ 100 mg/dL (17), high triglycerides (TG) ≥130 mg/dL(17), high total cholesterol (TC) ≥200 mg/dL, high LDL (low-density lipoprotein) ≥130 mg/dL, low HDL (high-density lipoprotein) ≤40 mg/dL, high ALT (alanin aminotransferase) ≥ 40 U/L(14), high AST (aspartate aminotransferase) ≥ 40 U/L (14), high SBP (systolic blood pressure) ≥ 130 mmHg, high DBP (diastolic blood pressure) ≥ 85 mm Hg, High SBP and/or DBP ≥ 130/85 mm Hg, MetS (metabolic syndrome) based on having 3 or more than 3 of the criteria above (TG, HDL, SBP and/or DBP, FBS, WC). high TG ≥150 mg/dL is another cut-point used here.^18^ ^1^ *P* values are resulted from chi-square test.

## Discussion

This study, which to the best of our knowledge is the first national study in developing countries that compared the mean and prevalence of cardio-metabolic risk factors and elevated liver enzymes in a pediatric population living in areas with different SES, demonstrated higher prevalence of risk factors in those living in higher than in lower SES areas. Individuals living in areas with higher SES, i.e. Central and Western part of Iran, were more likely to have adverse lipid profile, namely hypertriglyceridemia and low HDL-C, along with elevated liver enzymes and MetS, than their other counterparts.

Low HDL- C was the most frequent disorder affecting the study population ranging from 25.9% to 42.9%. We found a relatively high frequency of low HDL-C and elevated TG^22^ in the Western part (second high SES) of the country. Hypertriglyceridemia^22^ was the second prevalent disorder affecting 16.3% of residents from the West region (second high SES); ranging from 10.5% to 16.3% across the four pre-defined districts of Iran. Our findings are consistent with previous studies that have reported high rates of MetS, cardio-metabolic risk factors, and elevated liver enzymes in Iranian children and adolescents,^16,17,19,25-29^ however they did not assess the regional disparities. For instance, we previously found that ALT, total cholesterol, LDL-C, TG and SBP increased from lower to higher categories of BMI, and HDL-C decreased significantly. Increased levels of ALT, AST, and alkaline phosphatase were documented in respectively 9.5%, 9.8% and 9.1% of overweight subjects, and 16.9%, 14.9% and 10.8% of obese individuals.^20^ The first survey of the CASPIAN study revealed that those children with hypertriglyceridemic waist phenotype were more likely to have cardiovascular risk factors, especially overweight and hypercholesterolemia.^23^

Significant regional variations in the prevalence of cardio-metabolic risk factors such as hypertension, diabetes and dyslipidemia are also reported in adult populations. According to the National Health and Nutrition Examination Survey (NHANES), higher frequency of hypertension and metabolic risk factors were observed in the Southeastern states of America.^30^ European multisite studies such as the British Regional Heart Study have indicated diverse frequencies of risk factors in different regions of these countries. Higher rates in cardio-metabolic risk profiles have been reported in the North and East European countries compared to the Southern parts.^31,32^ Hypertension and hypercholesterolemia were more abundant in the Northeast regions of China as compared to others.^33^

Studies conducted among adult populations revealed that in most countries, people with a low SES have high rates of unhealthy lifestyle behaviors and insufficient health care, and in turn they face higher morbidity and mortality of NCDs and their risk factors.^3^ Controversial results are reported about the prevalence of cardio-metabolic risk factors in children and adolescents with different SES levels. In general, it can be assumed that in low- and middle-income countries, children with higher SES have higher prevalence of cardio-metabolic risk factors,^34,35^ whereas in high-income countries those with lower SES are at increased risk for these risk factors, which may persist across the life course.^36-38^

It is well established that NCDs in adulthood are highly correlated with the emergence of adverse risk factor levels early in life.^14^ The unfavorable profile of a population could help to predict future burden of chronic diseases and related morbidity and mortality.^22^ Although a combination of multiple factors including genetics, behavioral, cultural and environmental might affect body weight, one major contributor to health status is defined as SES. Definition and description of socio-economic class is varied between studies due to the different opinions of investigators and the parameters measured. The current study evaluated SES as an indicator consisted of geographical districts of Iran and social class of participants to show regional disparities.

Social inequalities in risk factors are suggested to be responsible for more than half of inequalities in major NCDs, and early childhood development programs and education are considered of effective interventions in this regard.^3^ The findings of our study propose that effective strategies on primordial and primary prevention of NCDs should also consider the higher frequency of risk factors in high SES groups of children and adolescents, which may be because of their sedentary lifestyle and Westernized food pattern.^39^ Our finding can be of help for policy makers to have interventions at sub-national levels; such interventions would not need individual assessment, and can be designed based on the current findings.

## Study limitations and strengths

The major limitation of the current study is its cross-sectional design; the main strength is the novelty in providing nationwide data from the pediatric population of developing countries, as well as assessment of biological and biochemical risk factors in a large population-based sample of adolescents.

This study is confirmatory evidence that in communities with large variations in health and SES in different regions, health policies for primordial and primary prevention of NCDs have to be made at sub-national levels. Our findings can be used by health policy makers in developing action-oriented programs for these health and social issues from early life. This study proposes forming policies on preventing cardio-metabolic risk factors in adolescents for regions with higher SES, while forming policies on early childhood development in regions with lower SES. We suggest increasing the sample size of future national surveys in a way to be able to have all these estimations not only at regional level, but also at provincial level.

## Acknowledgments

This nationwide study was conducted as a survey of a school-based surveillance program. The authors are thankful to the large team working with this study, as well as the participants and their families.

## Competing interests

The authors have no conflict of interest.

## Ethical approval

A comprehensive verbal description of the nature and purpose of the study was given to the students, their parents and teachers. Written informed consent was obtained from parents. Ethical committees of Tehran University of Medical Sciences and Isfahan University of Medical Sciences reviewed and approved study protocols.
